# Palliative Care in Inflammatory Bowel Disease: A New Partnership

**DOI:** 10.1093/crocol/otab062

**Published:** 2021-09-01

**Authors:** Emily B Rivet, Jaime L Bohl, Sarmed Al Yassin, Stephen J Bickston

**Affiliations:** 1 Department of Surgery, Division of Colon and Rectal Surgery, Section of Hospice and Palliative Medicine, Virginia Commonwealth University, Richmond, Virginia, USA; 5 Department of Internal Medicine, Division of Colon and Rectal Surgery, Section of Hospice and Palliative Medicine, Virginia Commonwealth University, Richmond, Virginia, USA; 2 Department of Surgery, Division of Colon and Rectal Surgery, Virginia Commonwealth University, Richmond, Virginia, USA; 3 Department of Gastroenterology, Virginia Commonwealth University, Richmond, Virginia, USA; 4 Department of Internal Medicine, Inflammatory Bowel Disease Center, Virginia Commonwealth University, Richmond, Virginia, USA; 6 Department Gastroenterology, Inflammatory Bowel Disease Center, Virginia Commonwealth University, Richmond, Virginia, USA

**Keywords:** palliative care, inflammatory bowel disease, total pain

## Abstract

**Background:**

Palliative care (PC) is being increasingly recognized for benefitting patients with a wide spectrum of chronic serious medical conditions.

**Methods:**

Care models and principles of PC for patient with inflammatory bowel disease were explored.

**Results:**

The use of a structured and systematic approach for emotionally laden conversations and the “Total Pain” paradigm are examples of PC expertise that can be applied through either primary or consultative PC models.

**Conclusions:**

PC should be considered in clinical practice and as a topic for further scholarly investigation to further define its role and benefits.

## Graphical Abstract

**Figure:**
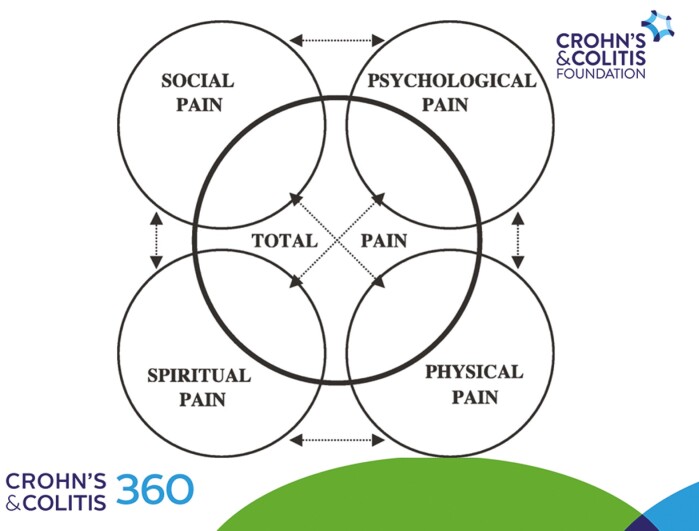

The discipline of palliative care (PC) is being increasingly recognized for its diverse benefits for patients with serious illness. Although Hospice and Palliative Medicine (HPM) was not defined as a medical specialty until 2006, the term PC was introduced by a Canadian Urologist, Dr Balfour Mount, in 1975.^1^ The principles of PC include relief of suffering, attention to psychosocial and spiritual needs, support for complex health-care decision-making and thoughtful communication. The unit of care in PC includes both the patient and caregiver. Although the evidence is mixed regarding whether patients with inflammatory bowel disease (IBD) have decreased life expectancy, these patients experience significant and complex symptoms, decreased quality of life, difficult health-care decision-making, and caregiver burden that aligns with the services of PC. Similarly, to patients with other chronic serious illnesses such as cardiac, pulmonary, neurologic, and kidney disease, patients with IBD are likely to benefit from access to PC.^2^

There are 2 structures for PC delivery. The first, *primary palliative care*, is provided in the course of usual care from the treating gastroenterologist, surgeon, or other specialist. This care can be enhanced by the education and training of these practitioners in PC principles and their application. The second is termed *consultative or specialty palliative care* and refers to care performed by a separate multidisciplinary team which generally includes a provider with advanced training and certification in HPM as well as experts in fields such as Social Work and Chaplaincy.

Despite the alignment between the needs of the patient population and expertise of the specialty, the role of PC in IBD has been largely unexplored. The last article focusing on PC for IBD patients was published more than 20 years ago and PC consultation for IBD patients is not standard clinical practice.^3^ The reasons for this are likely related to the historical development of PC in oncology. In IBD, the symptom burden across physical and psychological domains, and high resource utilization make the case for incorporating principles and tools of PC

Symptom management is a core expertise of PC. A patient suffering from pain, anxiety, or nausea cannot be reasonably expected to participate in a complex discussion about treatment options. One of the most common symptoms impacting patients with IBD is pain.^4^ The concept of “Total Pain” (TP) which was first introduced by Dame Cicely Saunders, the founder of the Hospice movement, provides a framework for understanding that the emotional, social, and financial burdens of an illness directly influence a patient’s physical pain.^5^ The relationship between physical and psychosocial pain can be self-perpetuating, with physical pain exacerbating anxiety and depression (see Figure 1). Specialists who focus on a single aspect of the total pain rubric may miss the interconnected nature of these factors. Although management of anxiety and depression may not be the focus of the medical and surgical specialties caring for IBD patients, an awareness of the importance of these factors can help prompt referral for treatment. The poor outcomes associated with corticosteroid and opioid use in this population highlight the importance of determining the cause of pain, appropriately treating it, and ensuring follow-up for durable maintenance regimens.

**Figure 1.**
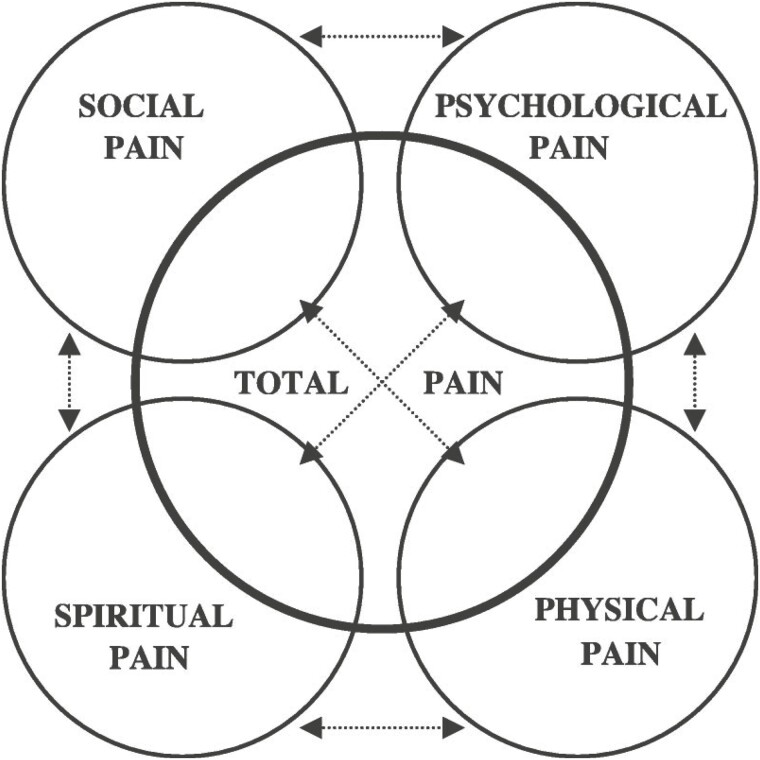
The complex interplay between types of suffering with the net result of “Total Pain” as described by Saunders.

Communication during the treatment of IBD patients can be emotionally laden. Complicated topics such as suggesting the option of a colostomy for anal Crohn’s disease can be taken as a “breaking bad news” discussion. Best practices for communication that are taught and practiced in palliative care can help avoid communication breakdowns between patients and the health care team. For example, consider the following application of the “SPIKES” protocol to this topic.^6^ (*S*)et up and prepare for the discussion by asking the patient who they would like to have present for the conversation. Some patients may prefer to have a spouse or other caregiver involved for support and advocacy, others may feel that this is a deeply personal subject that they prefer to learn about independently. Then, assess the patient’s (*P*)erspective by asking what they think or know about it. A patient may have done considerable research, having realized on their own that an ostomy might be a treatment option, while others will be very startled and upset at the introduction of this possibility. Next, get the patient’s (*I*)nvitation to talk. Sometimes a clinician might ask something like, “Would it be OK to talk about the option of a colostomy?” The fourth step in SPIKES is to provide (*K*)nowledge—it’s at this point that the potential clinical benefits and risks of the intervention can be discussed. And if the patient responds with emotion, (*E*)mpathy is appropriate, using statements like, “Patients I’ve cared for in the past have felt scared when they first thought about a colostomy as an option.” Lastly, provide a (*S*)ummary and strategy for next steps. As stated, SPIKES is one of the many protocols for delivering bad news; the aim of providing this example is to demonstrate how standard clinical interactions in the care of IBD patients can benefit from considering these moments as events amenable to PC relevant skills.

While PC resources vary among institutions, primary and consultative primary care models provide distinct options for integrated care. The fact that access to PC can be provided by either the usual treating team or via consultation should be emphasized. Embedded models in which a PC specialist has an outpatient presence in a specialty clinic are an increasingly popular option.^7^ However, another alternative is to provide education to the existing IBD team to enhance their skillset. Integrating the Total Pain conceptual model and using a structured approach like SPIKES for difficult conversations are only two of many examples in which a PC approach can benefit patients with IBD. PC is being increasingly leveraged beyond oncology to improve care for a variety of patient populations. These include patients with cardiac, pulmonary, neurologic, and kidney conditions. We encourage clinical use of PC and further scholarly investigation of its role in IBD.

## Data Availability

No new data were created or analyzed.

## References

[1] Miller P . Surgical palliative care – where are we in 2020? Am Surg. 2020;86(11):1436–1440. 33231479 10.1177/0003134820965951

[2] Quinn KL , Shurrab M, Gitau K, et al. Association of receipt of palliative care interventions with health care use, quality of life, and symptom burden among adults with chronic noncancer illness: a systematic review and meta-analysis. JAMA. 2020;324(14):1439–1450. 33048152 10.1001/jama.2020.14205 PMC8094426

[3] Gerson LB , Triadafilopoulos G. Palliative care in inflammatory bowel disease: an evidence-based approach. Inflamm Bowel Dis. 2000;6(3):228–243. 10961595 10.1097/00054725-200008000-00009

[4] Zielińska A , Sałaga M, Włodarczyk M, Fichna J. Focus on current and future management possibilities in inflammatory bowel disease-related chronic pain. Int J Colorectal Dis. 2019;34(2):217–227. 30564910 10.1007/s00384-018-3218-0 PMC6331746

[5] Clark D . ‘Total pain’, disciplinary power and the body in the work of Cicely Saunders, 1958-1967. Soc Sci Med. 1999;49(6):727–736. 10459885 10.1016/s0277-9536(99)00098-2

[6] Baile WF , Buckman R, Lenzi R, Glober G, Beale EA, Kudelka AP. SPIKES-A six-step protocol for delivering bad news: application to the patient with cancer. Oncologist. 2000;5(4):302–311. 10964998 10.1634/theoncologist.5-4-302

[7] Rhee C , McHugh M, Tun S, et al. Advantages and challenges of an interdisciplinary palliative care team approach to surgical care. Surg Clin North Am. 2019;99(5):815–821. 31446910 10.1016/j.suc.2019.05.004

